# Management of severe and fulminant Clostridioides difficile infection in adults

**DOI:** 10.1099/jmm.0.001991

**Published:** 2025-04-24

**Authors:** Daisy Ubsdell, Nicola Louise Maddox, Ray Sheridan

**Affiliations:** 1 Royal Devon University Healthcare NHS Foundation Trust, Exeter, UK; 2 Royal Devon University Healthcare NHS Foundation Trust and North Bristol NHS Trust, Bristol, UK

**Keywords:** adults, *Clostridioides difficile*, *C. difficile* infection, severe

## Abstract

*A corrigendum of this article has been published full details can be found at* *https://doi.org/10.1099/jmm.0.002062*

*Clostridioides difficile* (formerly known as *Clostridium difficile*) is a significant cause of healthcare-associated infection with symptoms ranging from diarrhoea and abdominal pain to pseudomembranous colitis and toxic megacolon. Severe disease can pose a significant morbidity and mortality risk and is to be considered a medical emergency. The emergence of a new *C. difficile* ribotype with an estimated mortality rate of 20% (ribotype 995) has prompted a re-review of the evidence and guidelines around managing severe *C. difficile* infections (CDI). International guidance on the management of CDI varies regarding first-line antibiotic choice. Metronidazole is no longer favoured as first line due to concerns around resistance, and vancomycin and fidaxomicin are now recommended as first line options. Antibiotic therapy should be used in conjunction with good supportive measures and early consideration of surgical management. Faecal microbiota transplant can be utilized in recurrent CDI and may be useful in severe disease. Severe CDI is a significant ongoing threat to public health, and further research into effective management is essential to ensure the best possible outcomes for patients.

## Introduction

*Clostridioides difficile* (formerly known as *Clostridium difficile*) is a gram-positive and spore-forming bacterium [1]. The *C. difficile* spores facilitate the survival and transmission of this bacteria in the environment by adhering to surfaces and soil [2,3]. This obligate anaerobic bacillus is transmitted via the faecal-oral route and can colonize the human intestinal tract without causing infection. However, healthcare facilities can become reservoirs for *C. difficile* spores and risk patient infection if there is a lack of adherence to cleaning protocols. It is estimated to be present in the gut flora of <2% of UK adults without recent healthcare contact but can reach up to 26% of adult inpatients in acute care hospitals [3,5].

*C. difficile* infection (CDI) occurs when this bacterium proliferates and secretes toxins, namely Toxin A and Toxin B, with Toxin B being the more potent virulence factor [5]. This causes intestinal epithelium damage leading to diarrhoea, colitis, abdominal pain, fever and distension [6,7]. The principal CDI risk factor is broad-spectrum antibiotic usage which causes disruption of the normal gut flora, allowing overgrowth of *C. difficile* and colonization with more pathogenic variants [4,6, 8]. CDI ranges from asymptomatic carriage to severe and potentially fatal conditions such as pseudomembranous colitis and toxic megacolon. It is one of the most commonest healthcare-associated infections (HCAI), particularly in older hospitalized patients, but is becoming increasingly recognized in community settings with 20–30% of cases of CDI now considered to be community associated [1,9, 10]. It has significant morbidity, and 30-day all-cause mortality is estimated to be between 9 and 38% [11]. Severe and life-threatening CDI should be considered a medical emergency.

*C. difficile* is classified into strains via polymerase chain reaction (PCR) ribotyping (RT). Toxigenic *C. difficile* strains generally produce both Toxin A and Toxin B. However, some toxigenic strains are Toxin A negative, Toxin B positive due to gene mutations; RT017 is an important example given it can cause severe CDI equivalent to dual Toxins A and B producing strains [12]. In the last two decades, a hypervirulent, antibiotic-resistant, epidemic *C. difficile* strain emerged; RT027 [13]. This was felt to contribute to the increased incidence and severity of CDI in North America and Europe with large outbreaks of severe, often fatal, colitis. Notably, there was a sixfold increase in CDI-associated mortality from 1999 to 2006 in England and Wales [2,14, 15]. NHS England implemented surveillance programmes, measures to control antibiotic prescribing, patient isolation and hygiene protocols which led to a significant decrease in cases from 2007/08 to 2013/14 (falling from 107.6 cases per 100 000 population to 24.8 cases per 100 000 population) [4]. Subsequently, there was a decrease in CDI caused by RT027 in Europe, most notably in the UK [16].

However, in 2021/2022, UK CDI cases started to increase. The UK Health Security Agency (UKHSA) is currently investigating a new ribotype (995) which has appeared in the UK over the last 2 years [17]. There is a suggestion that RT995 is potentially similar to RT027 following detection of a deletion in the toxin regulator and microbiological fingerprinting showing the isolates are very closely related. Many patients have been presenting clinically with severe disease, and there is an associated significant mortality, approximately 20% to date. It has caused several large hospital clusters which were difficult to control, and it appears to be quick to transmit, with some younger patients affected. Importantly, it has been found to be resistant to metronidazole on sensitivity testing, one of the key antimicrobial agents for treating CDI. UK healthcare professionals must be vigilant to the emergence of this new ribotype, especially when managing healthcare-associated diarrhoea outbreaks. Subsequently, metronidazole should not be used as first-line treatment [17].

CDI evidently remains an evolving field, and the current concern is that this new circulating ribotype 995 or future strains will lead to increasing cases of severe or fulminant CDI. This review focuses on the management of severe and fulminant CDI in adult patients, with a particular focus on future treatments, including faecal microbiota transplant (FMT) and probiotics, given the public interest in these new therapies.

## Initial assessment of CDI

### Diagnosis

CDI should be clinically suspected when patients present with a new onset of three or more unformed stools within 24 h, especially in the context of recent antibiotics and healthcare setting exposure [18].

Appropriate laboratory testing of a stool sample should be undertaken to confirm the suspected diagnosis. There are four main tests used to diagnose CDI which are detailed in Table 1 alongside the advantages and disadvantages [19]. False positives may occur in the presence of bloody diarrhoea.

**Table 1.** Laboratory tests for CDI, adapted from U.S. Centres for Disease Control and Prevention, Clinical Testing and Diagnosis for CDI

	Advantages	Disadvantages
**Molecular tests**	Normally a PCR assay which is a same day test. It is highly specific and sensitive for a toxin-producing *C. difficile* organism.	Can be positive in asymptomatic individuals or those with another pathology which can lead to over-diagnosis and treatment.
**Antigen tests**	Rapid test (<1 h) which detects the presence of *C. difficile* antigen glutamate dehydrogenase (GDH).	Antigen testing alone is non-specific and needs to be combined with tests for toxin detection.
**Toxin tests**	Specific and sensitive results for CDI. N.B. less sensitive than PCR or toxigenic culture.	Tissue culture cytotoxicity assay only detects Toxin B. Requires technical expertise to perform, is expensive and takes 24–48 h for a result. The *C. difficile* toxin is unstable and can lead to false-negative results if the sample is processed slowly.
**Stool culture**	The most sensitive test available.	Labour-intensive and slow to grow. Results take 48–96 h. Requires an appropriate culture environment. Can result in false positives.

It is suggested that either a one-step process (molecular test) or multi-step process (e.g. antigen plus toxin test) is undertaken to test for CDI [18]. It is important to note that whole genome sequencing (WGS) is gaining traction as a future diagnostic approach for CDI. This has the benefit of being able to differentiate a relapse of infection (with the same strain) versus a new infection (with a different strain), which can help personalize treatment and management [20].

### Inpatient versus outpatient treatment

CDI can occur in both community and hospitalized patients. Severe or fulminant CDI should be considered a medical emergency and requires prompt hospital admission if not already an in-patient.

Once a patient has confirmed CDI on stool testing and it is deemed to be severe or fulminant, they need an urgent clinical review, a NEWS2 score and blood tests (full blood count (FBC), urea and electrolytes (U&Es), C-reactive protein (CRP), albumin). Abdominal imaging should be considered. The patient should be isolated in a side-room with infection control precautions in place (gloves, apron, handwashing with soap and water). A severity assessment and mortality scoring should be undertaken alongside early referrals to specialist teams including but not limited to microbiology, infection control and the specialist medical team. An early surgical opinion should be obtained for the consideration of surgical management in patients with evidence of colitis.

### Risk factors

The main risk factor for CDI is antibiotic usage, with patients being 7 to 10 times more likely to acquire CDI whilst on antibiotics and in the month after [21]. Prolonged or multiple courses of antibiotics, especially fluoroquinolones, disrupt the gut flora allowing overgrowth of pathogenic strains of *C. difficile*. For patients with suspected or confirmed CDI, existing antibiotic treatment should be reviewed and stopped unless essential [8].

A UK national stewardship intervention was established to reduce the use of the 4C antibiotics which are associated with CDI; ciprofloxacin (fluoroquinolones), clindamycin, co-amoxiclav and cephalosporins. One study showed this intervention resulted in substantial declines in total CDI [22]. A 2016 systematic review and meta-analysis of antibiotics and CDI found that clindamycin and carbapenems were associated with more CDIs than other antibiotics [23]. The 2024 UKHSA data reflects that co-amoxiclav and piperacillin/tazobactam have the highest association with CDI [24]. The 4C was a very useful prompt, but given the latest data, we suggest that this could be updated to 5C + T (with the fifth C being carbapenems and T being piperacillin/tazobactam).

In addition, for people with suspected or confirmed CDI, review the need to continue any treatment with:

- Proton pump inhibitors;
- Other medicines with gastrointestinal activity or adverse effects, such as laxatives;
- Medicines that may cause problems if people are dehydrated, such as non-steroidal anti-inflammatory drugs, angiotensin-converting enzyme inhibitors, angiotensin-2 receptor antagonists and diuretics;
- Loperamide should be avoided due to the potential risk of toxic megacolon [25].

Other risk factors include older age (65 or older), frailty, immunosuppressed patients, recent healthcare stay (hospitals and nursing homes), previous CDI or known exposure [1, 8]. In those patients who are more susceptible to CDI, the risk of developing more severe disease may be higher [26,27]. There is potential for the development of vaccines to prevent CDI or reduce the severity of disease for those at high risk. A recently published phase III trial suggested that a *C. difficile* recombinant toxin vaccine showed potential to prevent progression to severe disease; however, there are no currently licenced vaccines [28].

### Relapse versus recurrence

For the first episodes, patients should receive initial antimicrobial management for CDI. For further episodes, there are other treatment considerations (which are discussed later in this review). However, it is recognized that it is difficult to distinguish whether a further episode is relapse or recurrence. There is no consensus on the precise definition, but the National Institute for Health and Care Excellence (NICE) 2021 guidance suggests the following descriptions [29]:

- *Relapse*: occurs within 12 weeks of previous symptom resolution.
- *Recurrence*: occurs more than 12 weeks after previous symptom resolution.

### Fluid losses and other supportive measures

Patients with CDI are at risk of dehydration and need strict hydration management. Clinicians need to manage fluid loss and symptoms of acute gastroenteritis. Patients should not be given antimotility medicines such as loperamide.

Patients should also have a risk assessment for venous thromboembolism and be given prophylaxis if not contra-indicated. Physiotherapy input may be required to enable early remobilization [29].

### Reassessment

CDI patients should be closely monitored, whether this be in the community or hospital. They need an urgent reassessment if the patient does not improve with initial management or if they deteriorate. Ideally, hospital inpatients should be reviewed daily by clinicians [29].

## Classifying CDI severity

### Case definition

There is not an internationally agreed case definition of CDI disease severity. Tables2^5^ summarize the key international bodies' guidance for CDI severity grading [30].

**Table 2.** NICE Guidance 2021 [29] – Severity of *C. difficile* infection [29]

**Mild infection**	Not associated with an increased white cell count (WCC). Typically associated with fewer than three episodes of loose stools (defined as loose enough to take the shape of the container used to sample them) per day.
**Moderate infection**	Associated with an increased WCC (but less than 15×10^9^ l^−1^). Typically associated with 3 to 5 loose stools per day.
**Severe infection**	Associated with a WCC greater than 15×10^9^ l^−1^ or an acutely increased serum creatinine concentration (greater than 50% increase above baseline), or a temperature higher than 38.5 °C, or evidence of severe colitis (abdominal or radiological signs). The number of stools may be a less reliable indicator of severity.
**Life-threatening infection**	Symptoms and signs include hypotension, partial or complete ileus, toxic megacolon or CT evidence of severe disease.

**Table 3.** IDSA Guidance 2021 – Severity of *C. difficile* infection [31]

**Non-severe**	Leukocytosis with a WCC less than 15×10^9^ l^−1^ and a serum creatinine level <1.5 mg dl^−1^
**Severe**	Leukocytosis with a WCC greater than 15×10^9^ per litre cells ml^−1^ or a serum creatinine level >1.5 mg dl^−1^
**Fulminant**	Hypotension or shock, ileus and megacolon

**Table 4.** ESCMID 2021 - Severity of *C. difficile* infection [32]

**Non-severe**	WBC count of ≤15×10^9^ l^−1^ and a serum creatinine level ≤50% above baseline, and core body temperature at presentation ≤38.5 °C. No imaging features of severity.
**Severe**	Characterized by one of the following factors at presentation: fever, i.e. core body temperature >38.5 °C, marked leukocytosis, i.e. leucocyte count >15×10^9^ l^−1^, and rise in serum creatinine, i.e. >50% above the baseline. Additional supporting factors, when available, are distension of the large intestine, peri-colonic fat stranding or colonic wall thickening (including low-attenuation mural thickening) at imaging.
**Severe-complicated (or fulminant)**	Defined by the presence of one of the following factors that needs to be attributed to CDI: hypotension, septic shock, elevated serum lactate, ileus, toxic megacolon, bowel perforation or any fulminant course of disease (i.e. rapid deterioration of the patient).

**Table 5.** ASID 2016 - Severity of *C. difficile* infection [33]

**Non-severe**	Absence of all features consistent with severe CDI.
**Severe**	Any of the following features if no other explanation can be provided: WBC count of >15 000 cells ml^−1^ or a rise in serum creatinine level >50% above baseline or core body temperature >38.5 °C. Rigours, haemodynamic instability, peritonitis or evidence of bowel perforation, ileus or toxic megacolon, elevated lactate level, albumin level <25 mg l^−1^, large intestine distension, colonic wall thickening, fat stranding, unexplained ascites (imaging) or pseudomembranous colitis on colonoscopy.
**Severe-complicated (or fulminant**)	An episode of CDI complicated by toxic megacolon, admission to intensive care for severe sepsis, requirement for surgery or death due to CDI.

The NICE 2021 guidance for CDI categorizes cases into mild, moderate, severe and life-threatening diseases (Table 2) [29].

The Infectious Disease Society America (ISDA) 2021 CID guidelines categorizes cases as non-severe, severe and fulminant (Table 3) [31].

The European Society of Clinical Microbiology and Infectious Diseases (ESCMID) 2021 update on the treatment guidance document for CDI in adults categorizes cases as severe and severe-complicated (or fulminant) [32].

The Australasian Society for Infectious Diseases (ASID) 2016 guideline for the diagnosis and treatment of CDI categorize cases as non-severe, severe and severe-complicated (or fulminant) [33].

### CDI scoring tools

It can be difficult for non-specialists to accurately classify CDI severity using the above current classifications which are unvalidated and subjective. Clinical scoring systems are used in many areas of healthcare and can be useful, quick tools to classify disease severity, communicate this to other teams and predict outcomes. However, they do not negate the requirement for clinical judgement to be used in managing severely unwell patients. We discuss two validated scoring systems for CDI: the ALTAS scoring system and a prediction rule for risk of mortality in CDI.

The ATLAS scoring system was created to help non-specialists predict response to therapy in patients with CDI, including mortality rate [13, 34]. Table 6 summarizes the variables patients are scored against and the calculated cure rate and mortality rate based on points accrued.

**Table 6.** ATLAS scoring tool [34]

Variable	0 Points	1 Point	2 Points
Age (years)	<60	60–79	80 and greater
Treatment with systemic antibiotics during CDI therapy (≥1 day)	No	n/a	Yes
Temperature (°C)	37.5 or less	37.6–38.5	38.6 and greater
Leucocyte count (total)	<16 000	16 000–25 000	>25 000
Serum albumin (g/l)	>35	26–35	25 and less
Serum creatinine (μmol/l)	120 and less	121–179	180 and greater

ATLAS score (points)	Cure rate (%)	Mortality rate (%)
0	95.7	0.0
1	93.3	0.0
2	92.7	0.0
3	89.5	3.6
4	81.1	4.2
5	76.1	8.7
6	85.7	10.9
7	50.0	14.3
8	55.6	56.0
9	33.0	>56
10	<33.0	>56.0

There are limitations to the ATLAS scoring system. Extremely ill CDI patients (defined as life-threatening or fulminant) were excluded from analyses, which meant there was an under-representation of CDI patients in the upper extremes of the score values. Additionally, metronidazole treatment was not evaluated, and it remains unknown how the ATLAS score would perform in predicting cure rates with this antimicrobial [35]. However, the ATLAS score was evaluated against the IDSA 2017 CID guideline with the conclusion that it may be useful for evaluating CDI severity and determining drug therapy selection [36].

The Royal Devon University Healthcare (RDUH) NHS Foundation Trust (formerly Royal Devon and Exeter NHS Trust) and University of Exeter Medical School (UEMS) created a simple prediction rule, described in Table 7, deriving four from an original 186 variables (CRP, serum albumin, respiratory rate, WCC) to identify CDI patients' 30-day mortality risk. External validation of the tool against independent data from 158 patients was found to be effective if variables were measured within 48 h of CDI diagnosis [37].

**Table 7.** All-cause 30-day mortality scoring tool developed by Butt *et al*. [37]

Variable	Score (points)
Serum Albumin level ≤ 24.5 (g l^−1^)	1
CRP level > 228 (mg l^−1^)	1
WCC > 12 (mcL) and respiratory rate > 17 (resps min^−1^)	1

Total score (points)	Percentage mortality risk (derivation cohort):	Percentage mortality risk (validation cohort):
0	10.4%	20.9%
1	23.3%	37.1%
2	42.9%	54.3%
3	100%	66.7%

## Multidisciplinary team approach to managing severe/fulminant CDI

### Team members

CDI treatment, prevention and eradication requires a cohesive multidisciplinary team (MDT) approach in both an organizational context and an individual patient basis [38]. Patients with CDI require input from MDT that may include a microbiologist, infectious diseases specialist, infection control team, gastroenterologist, surgeon, pharmacist or dietitian, as needed [29].

Infection prevention and control specialists are vital in the management and prevention of CDI, not only for their clinical duties preventing the spread of CDI to other hospitalized patients but also for producing CDI guidelines and protocols at both organizational and national levels [36,38]. The 2019 American World Society of Emergency Surgery (WSES) guidelines suggest an infection control ‘bundle’ to be utilized in the context of CDI which focuses on antimicrobial rationalization, personal and environmental hygiene and education [39].

The UKHSA recommends a SIGHT approach for managing suspected or confirmed cases of infectious diarrhoea (Table 8) [11].

**Table 8.** UKHSA SIGHT protocol [11]

**S**	Suspect that a case may be infective where there is no clear alternative cause for diarrhoea
**I**	Isolate the patient and consult with the infection prevention and control (IPC) team while determining the cause of the diarrhoea
**G**	Gloves and aprons must be used for all contacts with the patient and their environment
**H**	Hand washing with soap and water should be carried out before and after each contact with the patient and the patient’s environment
**T**	Test the stool using a two-step testing system, sending a specimen immediately

### Education of hospital staff

Although specialists are vital for managing CDI in the healthcare settings, the wider hospital staffing also has a significant role to play by adhering to infection control policies and undertaking good antimicrobial stewardship [29]. The Centres for Disease Control and Prevention (CDC) recommend education for all healthcare staff on prevention practices as an integral aspect of managing CDI [40]. In 2016, S. Waqar and team showed that by introducing education in the form of signs, posters, training and e-learning, their hospital was successful in reducing rates of healthcare-associated CDI [41]. The infection control team often plays a lead role in trust-wide CDI education.

## Antimicrobial therapy for the first episode of severe or fulminant/life-threatening CDI

We have summarized the initial recommended antimicrobial treatment for a first episode of severe or fulminant CDI based upon NICE 2021 [29], IDSA 2021 [31], ESCMID 2021 [32] and ASID 2016 [33] guidelines (Table 9). It is difficult to know the optimal antibiotic therapy for critically ill patients with CDI as this group is usually excluded from prospective (randomized) clinical trials. However, we have discussed the most up-to-date evidence for the main antimicrobial therapies used in daily clinical practice for severe and fulminant CDI: vancomycin, metronidazole, fidaxomicin and tigecycline.

**Table 9.** Summary of main international guidance for antimicrobial treatment for severe and severe complications of fulminant CDI

	NICE 2021 [29]	ESCMID 2021 [32]	IDSA 2021 [31]	ASID 2016 [33]
**Severe**	**First line:** Vancomycin 125 mg orally four times a day for 10 days. **Second line:** Fidaxomicin 200 mg orally twice a day for 10 days.	Fidaxomicin OR Vancomycin 125 mg PO 6 hourly for 10 days. If oral administration is not possible: local delivery ± adjunctive intravenous therapy (metronidazole or tigecycline).	Fidaxomicin standard dose OR vancomycin 125 mg PO 6 hourly for 10 days and adjunctive bezlotoxumab for primary CDI if other risk factors for recurrence (age ≥ 65 years, immunocompromised host) or if episode in prior 6 months.	**First line:** Vancomycin 125 mg four times a day orally for 10 days. **Second line (if unable to tolerate oral therapy):** NG vancomycin 125 mg four times a day AND IV metronidazole 500 mg TDS ± rectal tube vancomycin 500 mg in 100 ml N. saline TDS-QID.
**Severe-complicated, fulminant or life-threatening**	Vancomycin 500 mg orally four times a day for 10 days PLUS, metronidazole 500 mg intravenously three times a day for 10 days.	Fidaxomicin OR vancomycin Multidisciplinary approach with surgical consideration. Consider intravenous tigecycline and faecal microbiota transplantation when refractory.	Vancomycin 500 mg 6 hourly PO or by nasogastric tube and metronidazole 500 mg IV 8 hourly AND consider vancomycin per rectum if ileus present.	Vancomycin up to 500 mg 6 hourly PO or by nasogastric tube ± vancomycin per rectum plus metronidazole 500 mg IV 8 hourly.

### Vancomycin

Vancomycin is a tricyclic glycopeptide antibiotic which targets the cell wall with a bactericidal effect and is used to treat gram-positive bacteria [42]. It has limited systemic bioavailability when administered orally but achieves high concentrations in the stool and is therefore used to treat specific gastrointestinal infections, one being *C. difficile* [43]. Vancomycin can be used in CDI in oral or rectal preparations [36]. Vancomycin remains the first-line antibiotic recommended by NICE (given cost-effectiveness) and ASID for severe CDI; however, ESCMID 2021 and IDSA 2021 [31] guidelines recommend vancomycin or fidaxomicin as the first-line antibiotic choices for severe CDI. A 2018 systematic review and meta-analysis found that for non-severe CDI, fidaxomicin or vancomycin can be used as first-line therapy, but vancomycin has been shown to be more effective for severe disease [44].

There is limited evidence regarding the rationale for standard dose vancomycin (125 mg QDS) versus high dose vancomycin (250–500 mg QDS), and it appears to be based upon historic practice [45]. A 2020 retrospective cohort study compared standard dose vancomycin with high dose vancomycin and reviewed outcomes of cure and recurrence. There was not a statistically significant difference in cure rate between the two groups, with 70.5% of patients in the low dose group and 72.7% in the high dose group achieving partial or total cure, with 15.38 and 15.15% recurrence/readmission rates in the low and high dose groups respectively [46]. Despite the lack of evidence for high dose versus low dose vancomycin, NICE, ASID and ISDA guidance all recommend high dose for life-threatening/fulminant CDI.

Rectal vancomycin can also be used off-label as a treatment for severe or fulminant CDI. IDSA and NICE guidance advise considering rectal vancomycin with specialist guidance, particularly in the case of ileus. ESCMID and ASID guidance suggests that rectal administration of vancomycin may be used when oral administration is not possible. A small 2015 study of 24 patients in ICU with severe or life-threatening CDI who received rectal vancomycin, compared with 48 matched control patients who did not receive rectal vancomycin, showed no difference in the percentage of patients who ended up receiving surgery (16.7% for both groups) or who ultimately died 45.5% (rectal vancomycin) and 41.7% (no rectal vancomycin) [47]. A case series published in 2016 suggested that patients treated with rectal vancomycin had similar surgical and mortality outcomes and length of hospital stay to those treated with oral vancomycin but had a reduced mortality compared with predicted mortality based on severity score [48]. Caution should be exercised in extrapolating data from small studies with no randomization. However, in practice, delivery of vancomycin rectally in cases where distal colitis is present makes clinical sense in delivering antibiotics to the site of active disease.

Of note, for recurrent CDI, vancomycin can be administered as a tapered pulsed dosing regimen. NICE does not recommend this as a treatment option as they felt its use was limited to studies where there was co-administration of FMT [29]. However, ESCMID and IDSA guidelines advise that vancomycin in a tapered and pulsed regimen can be considered for the first or subsequent CDI recurrence. A 2017 study suggests that when used appropriately, tapered pulsed vancomycin can cause clinical improvement in 73.3% of patients with recurrent CDI [49]. A 2019 retrospective study of patients with recurrent CDI found similar results with a 74% cure rate for patients with recurrent CDI treated with a tapered pulse regimen of vancomycin, as exemplified in Table 10 [50].

**Table 10.** An example of a vancomycin taper regimen as per Gerding and team, 2018 [34]

Week of treatment	Oral vancomycin dose and frequency
**1**	125 mg four times a day
**2**	125 mg three times a day
**3**	125 mg two times a day
**4**	125 mg one time a day
**5**	125 mg once every other day
**6**	125 mg once every third day.

### Fidaxomicin

Fidaxomicin is a first-in-class 18-membered macrocyclic bactericidal antibiotic (RNA-polymerase inhibitor) with minimal systemic absorption but high faecal concentrations. It is a relatively new treatment for CDI having been approved for usage in the European Union and the USA in 2011 [51,52]. However, it is an expensive drug which has hindered its usage worldwide due to unclear cost-effectiveness relative to current treatment options. Of note, the medication is taken twice daily (as opposed to vancomycin which is four times daily), which is a reduced pill burden for patients. It should be used with caution for those with a known macrolide allergy [53].

ASID 2016 guidelines recommend fidaxomicin as a last resort in severe disease, once all other options have been exhausted. NICE 2021 [29] guidance recommends fidaxomicin as a second-line treatment for mild, moderate or severe CDI (if vancomycin is ineffective) and additionally in recurrent or relapsing CDI. However, ESCMID 2021 and IDSA 2021 [31] guidelines recommend fidaxomicin as first-line treatment for initial episodes of CDI (regardless of severity) and especially for cases with a high risk of recurrence.

A 2019 retrospective study compared the use of oral vancomycin with oral fidaxomicin (standard dosing regimen) and reviewed outcomes of clinical failure, recurrence and mortality in patients with first episode of severe CDI. There was no statistically significant difference in the clinical treatment failure rate or recurrence between the two groups in severe disease [54]. A 2024 study by Okhuysen *et al*. found that fidaxomicin appeared superior to vancomycin in the preservation of the microbiome but did not reduce rates of recurrence and had poor efficacy against ribotype 027 [55].

One 2011 clinical trial suggested a similar clinical cure rate between the two treatments but a 45% relative reduction rate of CDI recurrence when treated with fidaxomicin compared with vancomycin [56]; this was reaffirmed by Cornely *et al.* in 2012 [52]. This suggests fidaxomicin could be a more effective treatment for recurrent CDI.

Overall, the guidelines and evidence differ in their recommendations regarding the use of fidaxomicin for severe or fulminant CDI and in recurrent CDI. Choices should be made in conjunction with clinical judgement, patient-centred care and health economics.

Of note, fidaxomicin has been investigated as a pulsed dosing regimen which involves 20 doses being spread over a period of 25 days (200 mg BD on days 1–5, 200 mg OD on alternate days on days 7–25). The rationale for extending the dosing regimen is that there is likely to be a lower chance of microbiome disruption. It was compared with vancomycin and findings suggested that it was superior for sustained cure of CDI, with 70% of patients achieving clinical cure after 30 days with extended pulsed fidaxomicin compared to 59% of patients treated with vancomycin [57]. NICE 2021 [29] guidelines considered this but felt there was insufficient evidence to justify recommending an unlicensed treatment regimen over a licensed one. However, IDSA 2021 guidelines recommend this as first-line treatment for first CDI recurrence. ESCMID 2021 guidelines state that extended-pulsed fidaxomicin should be considered for an episode of CDI with an increased risk of recurrence, especially in elderly hospitalized patients. Further comparative trials of extended-pulsed fidaxomicin dosing to standard fidaxomicin dosing are likely needed to understand where best to implement this regimen.

### Metronidazole

Metronidazole is a nitroimidazole antibiotic. In the late 1990s, the U.S. National Public Health Agency, Centre for Disease Control (CDC), recommended that vancomycin usage should be reduced in hospitals given the emergence of vancomycin-resistant enterococcus [58]. This led to an increasing use of metronidazole as a first-line treatment for CDI [59]. However, there is conflicting advice from international bodies. The ASID 2016 guidelines advise the use of oral metronidazole as first-line antibiotic for non-severe episodes and intravenous metronidazole as second-line therapy for severe disease. However, ESCMID 2021 and IDSA 2021 [31] guidelines advise that oral metronidazole should only be used if vancomycin or fidaxomicin are not available, and additionally, ESCMID does not recommend routine addition of IV metronidazole in severe and severe-complicated disease. NICE 2021 [29] guidelines only advise the use of IV metronidazole if first and second-line antibiotics (vancomycin and fidaxomicin respectively) are ineffective.

Clinicians must take note of the *C. difficile* strain ribotype (995) which has appeared in the UK over the last 2 years. This ribotype has a significant mortality (approximately 20% to date) and the strain has been found to be resistant to metronidazole. In January 2024, UKHSA advised UK clinicians not to use metronidazole for CDI, and this remains under review [17]. Given this, it is important to review the evidence around metronidazole usage in severe and fulminant CDI [60,61].

A 2015 meta-analysis reviewed six studies (four randomized control trials and two cohort studies) regarding the efficacy of vancomycin and metronidazole for treating CDI. It found that the rate of initial clinical cure was significantly higher in severe CDI patients treated with vancomycin (81%) than metronidazole (68%). There was not found to be any statistically significant difference in recurrence rate between the two antibiotic-treated groups [59]. It was felt that treatment outcomes with intravenous metronidazole were poor as patients with severe CDI would have reduced blood flow to the colon and therefore, less transduction of metronidazole. Vancomycin was also associated with lower recurrence rates in those with severe CDI. Additionally, a 2018 meta-analysis concluded that metronidazole was inferior at achieving sustained clinical cure compared with fidaxomicin and vancomycin and should not be recommended for CDI treatment [44]. Overall, this suggests that metronidazole should not be used as first-line treatment for severe CDI and instead should be reserved for cases not responding to first or second-line therapies.

### Tigecycline

Tigecycline is a tetracycline antibiotic with suggested high faecal concentrations and a limited impact on gut microflora [62]. *In vitro* studies have shown that tigecycline effectively inhibits sporulation of *C. difficile* [63]. ASID 2016 guidelines suggest that for patients with severe CDI who are unable to take oral therapies or are at high risk of treatment failure, intravenous tigecycline may present a potential successful treatment option. This is reiterated in ESCMID 2021 guidelines, although they note the evidence is weak. A retrospective case series reviewing severe and life-threatening CDI suggested that following administration of tigecycline in combination with either vancomycin or metronidazole, there was an 80% clinical cure and an 85.7% cure rate with triple therapy [64]. However, these results are only based on seven case studies with a varying tigecycline course length. A further 2022 retrospective case series analysis reviewed 28 cases of CDI treated with tigecycline combination therapy compared with 140 cases not treated with tigecycline. 75% of cases classified as either severe or fulminant showed a higher unadjusted 30-day mortality rate in tigecycline treated cases (14.3%) as compared to those not treated with tigecycline (5.3%), but no significant difference in mortality following risk adjustment [65]. Given the weak evidence, further research is required to investigate tigecycline as an adjunctive treatment for severe CDI, especially in those who are nil by mouth.

## Other therapies for consideration in severe or fulminant CDI

### Faecal microbiota transplant

FMT (also known as a stool or poo transplant) is not a new concept. Historical documents, dating back to fourth century China, suggest that human stool was used in unwell patients to treat diarrhoea and constipation [66]. In 1958, FMT was resurrected in clinical practice and used to successfully treat pseudomembranous enterocolitis in four patients [67]. FMT has since gained momentum and is now a recognized treatment for relapsing and refractory CDI in multiple countries worldwide. It involves the transfer of minimally manipulated faeces in a liquid form from a healthy screen donor to the intestine of a patient through either an upper gastrointestinal route or into the large bowel via a lower gastrointestinal route. It is thought to work by restoring beneficial gut microorganisms, but further research is required to understand the full mechanism of action. However, FMT has associated risks including the theoretical concern of bacterial translocation (limited evidence to date) and failure to achieve cure with one study reporting a 10–20% failure rate. Cure failure has been linked to higher severity of disease and higher number of previous episodes of CDI [68]. The ethical implications of FMT must also be considered, and there should be strict regulation of FMT to safeguard patients and donors [69].

The international bodies differ in their viewpoint regarding FMT for CDI are summarized in Table 11 [70,72].

**Table 11.** Summary of main international bodies guidance regarding FMT for CDI

	FMT recommendation
**IDSA 2021 [** **31** **]**	FMT only for patients with multiple recurrences of CDI who have failed appropriate antibiotic treatments and where appropriate screening of donor and donor faecal specimens has taken place.
**ASID 2016**	Recommend FMT as a therapeutic option for second or subsequent CDI recurrence if all other therapy options have failed and there are no contraindications.
**ESCMID 2021**	FMT may be a rescue therapy for patients with refractory severe complicated CDI for whom surgery is not feasible and in-patients with severe complicated CDI who have deteriorated despite CDI antibiotic treatment and for whom surgery is not feasible. However, they feel this is based on weak evidence and should be on a case-by-case basis with a careful risk assessment and MDT discussion.
**NICE 2022 [** **70** **]**	Recommend FMT as an option for adults who have had two or more previous confirmed CDI episodes.
**American Gastroenterology Association (AGA) 2024**	Recommends the use of FMT in hospitalized patients with severe or fulminant CDI who have not responded to antibiotics in 2–5 days but should not be considered in those with bowel perforations, obstruction or immunocompromised status.
**British Society of Gastroenterology (BSG) and Healthcare Infection Society (HIS) 2024**	Consider FMT for a first recurrence of CDI or as an adjunct to antibiotics in refractory CDI. Offer FMT to all patients with two or more recurrences of CDI. Consider FMT earlier than after the second CDI recurrence for patients with severe, fulminant or complicated CDI who are not responding to antibiotic therapy.

A 2019 study investigated the efficacy of FMT following a 4–10 day course of oral vancomycin in patients with recurrent CDI, which was compared to patients treated with either vancomycin or fidaxomicin only. Treatment with oral vancomycin and FMT was superior for recurrent CDI compared to antibiotics only [73]. Additionally, a 2023 Cochrane review compared outcomes of recurrent CDI patients treated with donor FMT with those who received placebo, autologous FMT or antibiotics. For individuals without immunocompromise, FMT was likely to lead to a reduction in recurrent CDI. 40% of individuals in the control group achieved resolution of recurrent CDI compared with 77% in those treated with FMT. It did not comment on the efficacy of FMT in immunocompromised individuals [74].

In 2017, the European FMT working group published the conclusion of their consensus conference on the use of FMT in clinical practice. Following significant and thorough analysis of available evidence, they suggested that FMT has been shown to have resolution rates of 85–89.7% in those with recurrent CDI and should be utilized in clinical practice with appropriate regulation [75]. This appears to be echoed by subsequent international guidance as detailed above; notably [72]. The BSG/HIS have recommended that FMT should be recommended to all patients regardless of underlying health status. However, it should be noted that the overall rate of severe adverse events for FMT was 0.65% in a population which included those with comorbidities and immunosuppressed status. The BSG/HIS also recommended that FMT could be considered earlier to prevent surgery or palliation in life-threatening CDI cases not responding to antibiotics [72]. The benefits versus risks of FMT should be part of the patient selection process and consent process.

Overall, FMT is becoming a more widely used CDI treatment. In light of this, several international bodies have recommended that for CDI patients being considered for FMT, an MDT approach should be undertaken with a risk assessment and MDT discussion on a case-by-case basis.

### Bezlotoxumab

Bezlotoxumab is a monoclonal antibody that binds and neutralizes the *C. difficile* main virulence factor Toxin B (it has no effect on Toxin A) [7]. In clinical trials, intravenous bezlotoxumab (in addition to standard care) has been shown to be effective at achieving clinical cure in recurrent CDI with 67% of patients achieving clinical cure compared with 52% of patients receiving standard care. However, it showed no difference in managing first episodes of CDI [76]. ESCMID 2021 recommends bezlotoxumab is considered as an adjuvant treatment for recurrent CDI alongside standard-of-care antibiotics when fidaxomicin is not available or feasible. NICE 2021 [29] guidelines do not recommend bezlotoxumab based on cost-effectiveness. IDSA 2021 [31] recommends that for patients with a recurrent CDI episode within the last 6 months, bezlotoxumab is used as a co-intervention along with antibiotics rather than antibiotics alone. However, in patients with a history of congestive heart failure (CHF), bezlotoxumab should only be used when the benefit outweighs the risk. ASID 2016 guidelines do not comment upon it. There is limited evidence for the use of bezlotoxumab in severe disease, and guidelines do not make recommendations for its use specifically in severe disease.

### Ridinizalole

Ridinilazole is a novel narrow-spectrum antibiotic of the bis-benzimidazole class of antimicrobials which has been found to be highly active against *C. difficile* [77]. US National Surveillance of *C. difficile* isolates were analysed between 2020 and 2021. 300 isolates were identified, and ridinilazole was found to have good antimicrobial activity against them all when compared with vancomycin and fidaxomicin. It also maintained activity against hypervirulent strains and those that showed resistance to other anti-microbial agents [78].

Initial trials with oral ridinilazole showed low systemic levels of ridinilazole with high faecal concentrations and limited impact on the gut microbiome [79]. A recent phase III trial of ridinilazole compared with vancomycin showed a sustained clinical response rate of 73% with ridinilazole versus 70.7% with vancomycin. Additionally, ridinilazole resulted in a 53% reduction in recurrence compared with vancomycin whilst preserving microbiota diversity [55]. Ridinizalole is not currently a recommended treatment in guidelines. This latest phase III trial was only published in 2024 so future guideline updates will need to consider its place in future treatment algorithms, given its reduced relapse rate and preservation of microbiome diversity.

### Human intravenous immunoglobulin

Healthy individuals express antibodies (specific immunoglobulins) against *C. difficile* toxins A and B. The levels of protective antibodies decrease as people age, which is felt to correlate with a higher risk of developing CDI [80]. Interestingly, patients with recurrent CDI have been found to have lower levels of antibodies, but there is limited evidence to suggest human intravenous immunoglobulin (IVIg) is a useful treatment for CDI, with the majority of studies being animal based or small sample sizes in human trials [81,84]. One 2017 study showed that 41% of patients had a therapeutic response to IVIg and that protective anti-toxin A and anti-toxin B neutralizing antibodies were in patients’ serum following IVIg treatment, suggesting there could be a sustained response to IVIg [84]. Currently, NICE and ESCMID guidance does not recommend IVIg as a treatment for CDI, and ASID and IDSA do not comment upon it. This area needs further research.

### Rifaximin

Rifaximin is a rifamycin-derived antibiotic with poor systemic absorption but high faecal concentrations which has historically been used for CDI [85]. However, there is a high resistance rate in the range of 29.1–48.9% which has led to reduced use of this in clinical practice [86, 87]. Only the ASID 2016 guidelines continue to recommend CDI treatment with this agent and following metronidazole, vancomycin or fidaxomicin failure or where FMT may not be available or contraindicated

### Vedanta Biosciences VE303

VE303 is an orally administered bacterial consortium candidate which is being developed for high-risk CDI. Eight clonal human commensal bacteria strains have been selected from healthy stool samples for their ability to provide colonization resistance to *C. difficile*. A phase II trial of VE303 showed a 30% absolute risk reduction of recurrence of CDI within 8 weeks of previous CDI episode and is currently undergoing phase III clinical trials. VE303 could be an alternative to FMT for preventing recurrence of CDI by recolonizing the gut and preventing overgrowth of pathogenic *C. difficile* [88].

### Probiotics

There have been extensive studies into the use of probiotics for CDI, but there is currently insufficient evidence to support probiotic use in current clinical guidelines [29]. A 2017 Cochrane review did suggest that probiotics may be effective in the treatment of CDI when used in conjunction with antibiotic therapy (for patients not immunocompromised or severely debilitated), but ESCMID 2021 guidelines reported that probiotics may delay microbiome reconstitution [32,89].

## Surgical management

Surgery remains a vital consideration in severe or fulminant CDI. Patients with toxic megacolon, ileus or significant colitis may need to undergo surgery, and ESCMID 2021 [32] and NICE 2021 [29] guidelines suggest that patients presenting with life-threatening disease or those not responding to treatment should have an early surgical referral. There is a high mortality of up to 47% associated with surgical therapy for CDI [90]. Individuals with comorbidities and increased frailty are pre-disposed to poorer surgical outcomes compared to the healthy population, and this should be considered when deciding on the suitability of surgical candidates [91]. There is limited evidence regarding set criteria for who should be managed surgically, and this should be approached on a case-by-case basis with MDT discussion and risk assessment [92].

Surgical management includes total abdominal colectomy (TAC), subtotal colectomy or loop ileostomy (LI). TAC involves removing the whole colon and creating a stoma. Subtotal colectomy involves removing part of the colon and creating a stoma. LI is a less invasive procedure and involves the development of a temporary stoma and possible removal of a small section of colon [92]. A 2017 multicentre trial investigated mortality risk associated with TAC compared to LI. LI had fewer complications and reduced blood loss in the intraoperative period with similar re-operation rate, similar post-operative complications and reduced mortality in comparison to TAC [93]. The mortality rate was reported as 31.3% in TAC and 26.2% following LI [94]. Another 2021 study found there was similar mortality data for both procedures in the context of severe CDI, but there was a higher chance of ileostomy reversal compared with TAC [95]. While the choice of procedure has little impact on overall mortality, LI may risk the chance of re-operation and TAC remains the gold-standard surgical procedure for CDI [96].

## Severe CDI case study

A female patient in her 80s presented to hospital with 5 days of abdominal pain, diarrhoea, anorexia, lethargy, fever and associated delirium. Her medical history was notable for a hip replacement 3 months previously. On examination, she had abdominal distension and tenderness with guarding. Her mortality risk at 30 days was calculated to be 66% using the RDUH/UEMS simple prediction rule and 14.3% using the ALTAS scoring tool (see Table 12 for detailed breakdown).

**Table 12.** ATLAS score and The Royal Devon and Exeter NHS Trust simple prediction rule score

WCC	19.7×10^9^ l^−1^	
CRP	404 mg l^−1^	Butt *et al*. Scoring tool result: 66% 30-day mortality risk.
Albumin	24 g l^−1^	
Creatinine	92 μmol l^−1^	ALTAS result: 50% cure rate, 14.3% mortality
Respiratory rate	21	

**Fig. 1.**
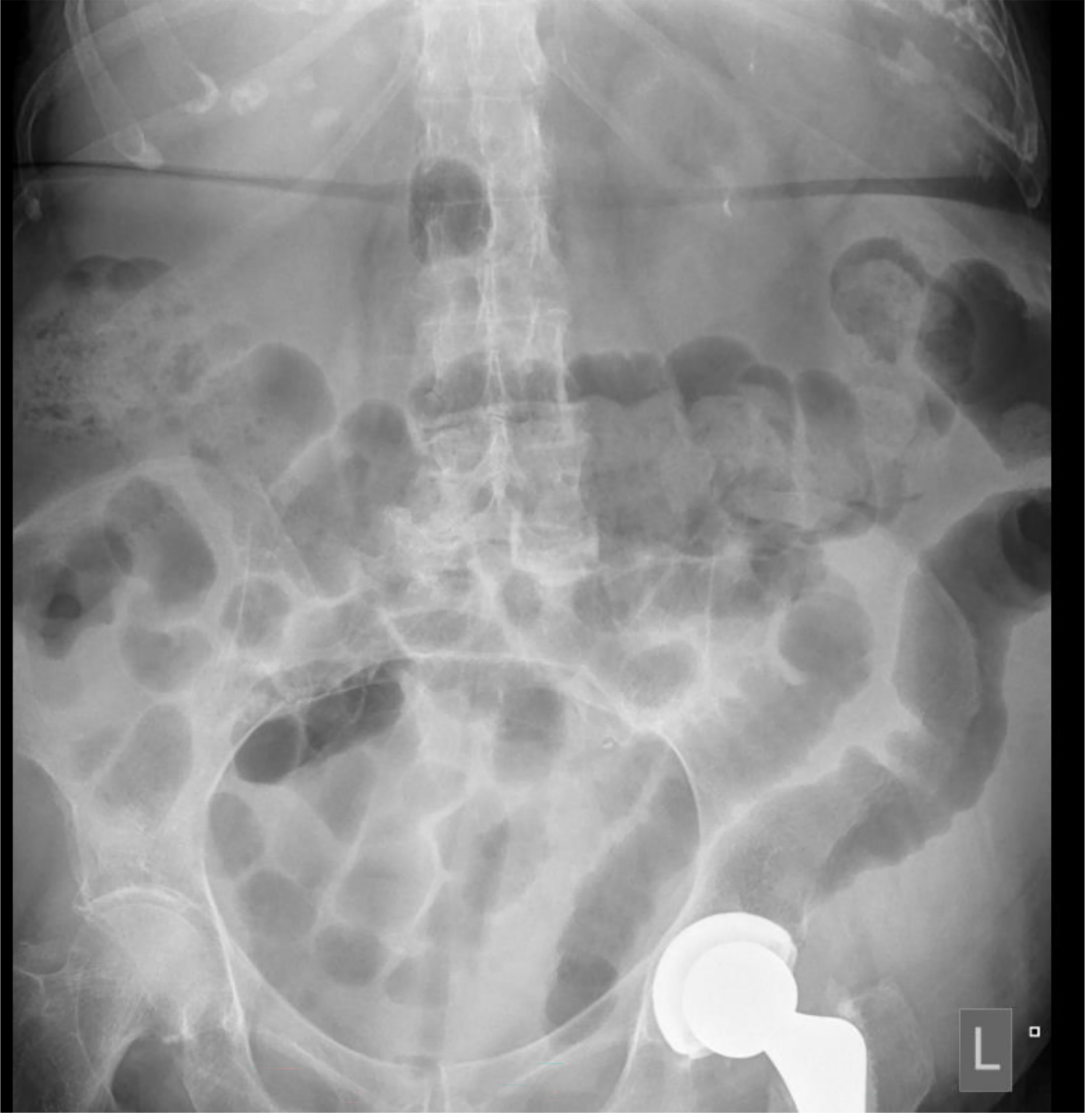
Abdominal X-ray showing evidence of colitis in the descending and sigmoid colon.

**Fig. 2.**
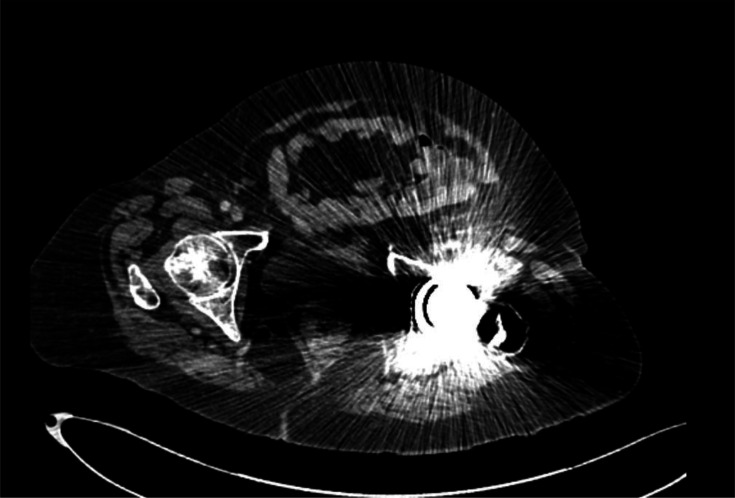
CT abdomen/pelvis showing evidence of colitis in the sigmoid colon and inflammatory changes.

She was commenced on intravenous antibiotics for a possible bacterial intra-abdominal infection, prior to knowing *C. difficile* status. An abdominal X-ray showing evidence of colitis in the descending and sigmoid colon (Fig. 1). A CT abdomen and pelvis completed on first admission was suggestive of acute sigmoid colitis or diverticulitis (Fig. 2). A stool sample subsequently confirmed *C. difficile*, toxin positive.

She was managed as severe CDI with colitis. IV antibiotics were switched to oral vancomycin 500 mg QDS and intravenous metronidazole 500 mg TDS for 10 days. Several days into admission, the patient deteriorated. A repeat CT abdomen and pelvis showed progression of colitis. Oral fidaxomicin and rectal vancomycin were added as adjunctive treatments. The patient stated that she wished to avoid surgical management of CDI. She improved clinically with medical management and was discharged after a 17-day admission.

Unfortunately, within 2 weeks of discharge, the patient began experiencing diarrhoea and abdominal pain again and was admitted to hospital for treatment of a relapsed CDI. She was managed with a 6-week tapering course of oral vancomycin plus 3 days of rectal vancomycin. She was discharged from hospital with ongoing oral vancomycin tapering and a plan for an outpatient FMT with the aim of preventing further relapses.

While awaiting FMT, she had a second relapse of CDI. She was admitted to hospital for the third time and commenced on oral and rectal vancomycin. FMT was given via an NJ tube and vancomycin was stopped. At the time of writing, the patient has not had any further relapses of CDI.

### Patient perspective

‘I don’t know how it all started because I was hallucinating terrible, really bad. I was put in an ambulance, and I just don’t remember why I was going away but when I finally found out what was going on, I was pooing throughout the night. It is quite scary, especially after just having my hip done. [It was explained to me that] Even with an operation, it would have been a big operation, so I took the chance and said no [to surgery] just do whatever you can to get me better, which he did. I felt a bit lonely being stuck in a room where nobody else was allowed, but I just got on with it, you know, and finally, the consultant said I was going to have some new poo. With that poo transplant, I thought it was marvellous, how it was done it was unbelievable, not a bit how I thought it was going to be. I never yelped or anything, I felt everything that was being done, and I got through that wonderfully.’

## Royal Devon University Healthcare NHS Foundation Trust management of severe CDI

International guidelines vary in their recommendations for the management of severe CDI and should be used in combination with clinical judgement. Here, we present a suggested algorithm used in our hospital (Table 13), considering evidence and international guidelines. It is pertinent to consult local and trust guidelines when managing a case of severe CDI as there will be variation between centres as to whether vancomycin or fidaxomicin will be first line.

**Table 13.** Suggested algorithm for managing severe CDI, ammended from the Royal Devon University Healthcare NHS Foundation Trust *C.difficile* guidelines

**Identification and isolation**	Clinical examination Isolate in side room Infection control measures
**Testing**	Stool sample testing
**Supportive management**	Hydration Rationalize medications Analgesia
**Investigations**	Observations and NEWS2 scoring Blood testing including CRP, albumin, white cell count Severity assessment Mortality scoring (as per Butt *et al*. or ATLAS) Imaging: X-ray abdomen and CT abdomen and pelvis Stool chart
**Referral**	Referral to microbiology team, specialist medical team Early referral to surgical team especially in the case of ileus, toxic megacolon, peritonitis or evidence of severe colitis on CT.
**Antibiotic therapy**	**Severe – Life-threatening:** First line: Oral Vancomycin 125 mg four times a day for 10 days Second line: Oral Fidaxomicin 200 mg twice a day for 10 days Refractory disease: Low threshold for escalating to: High dose oral Vancomycin 500 mg four times a day for 10 days IV Metronidazole 500 mg three times a day for 10 days Evidence of distal colitis: Rectal vancomycin **Recurrent CDI:** Fidaxomicin Repeat same course of initial antibiotics Tapering course of vancomycin Consideration of FMT with MDT discussion

## Conclusion

CDI remains a significant threat to patients and healthcare systems. Despite advances in the field, new concerning *C. difficile* strains are appearing. The recently identified ribotype 955 circulating in the UK prompted a re-review of current guidance for severe and fulminant CDI given the phenotype, metronidazole resistance and high mortality rate.

There are subtle discrepancies in the international guidelines, but there is consensus that severe, life-threatening or fulminant CDI is considered a medical emergency and should be managed accordingly. Clinicians should remain vigilant for severe and fulminant CDI and look to promptly review and treat these cases given the associated high mortality. The main international guidelines for treatment of severe and fulminant CDI differ on choice of first-line antibiotic therapy, but oral vancomycin and fidaxomicin are the mainstay of treatment, with FMT being considered in specific cases. There is the promise of new agents such as ridinilazole and Vedanta Biosciences VE303, but these need further research and clinical trials. Given the increasing elderly and co-morbid population, treatment decisions should be made in combination with clinical judgement following an MDT and patient-centred approach, especially when considering surgical intervention.

## Supplementary material

10.1099/jmm.0.001991 Uncited Supplementary Material 1.

## Abbreviations

ASID: Australasian Society for Infectious Diseases
CDC: Centres for Disease Control and Prevention
CDI: *C. difficile* infections
CHF: congestive heart failure
CRP: C-reactive protein
ESCMID: European Society of Clinical Microbiology and Infectious Diseases
FBC: full blood count
FMT: faecal microbiota transplant
GDH: glutamate dehydrogenase
HCAI: healthcare-associated infections
IPC: infection prevention and control
ISDA: Infectious Disease Society America
IVIg: intravenous immunoglobulin
LI: loop ileostomy
MDT: multidisciplinary team
NICE: National Institute for Health and Care Excellence
PCR: polymerase chain reaction
RDUH: Royal Devon University Healthcare
RT: ribotyping
TAC: total abdominal colectomy
UEMS: University of Exeter Medical School
U&Es: urea and electrolytes
UKHSA: UK Health Security Agency
WGS: whole genome sequencing
