# Corrigendum: Management of severe and fulminant Clostridioides difficile infection in adults

**DOI:** 10.1099/jmm.0.002062

**Published:** 2025-08-19

**Authors:** Daisy Usbell, Nicola Louise Maddox, Ray Sheridan

**Affiliations:** 1 Royal Devon University Healthcare NHS Foundation Trust, Exeter, UK; 2 Royal Devon University Healthcare NHS Foundation Trust and North Bristol NHS Trust, Bristol, UK

**Keywords:** C. difficile infection, Clostridioides difficile, adults, severe

In the published version of this article the ribotype 955 was incorrectly referred to as ‘ribotype 995’ for the majority of the article. The corrected text from the abstract and main body is presented below. The authors apologise for any inconvenience caused.

Within the abstract:

‘The emergence of a new *C. difficile* ribotype with an estimated mortality rate of 20% (ribotype 955) has prompted a re-review of the evidence and guidelines around managing severe *C. difficile* infections (CDI).

Within paragraph four of the introduction:

‘However, in 2021/2022, UK CDI cases started to increase. The UK Health Security Agency (UKHSA) is currently investigating a new ribotype (955) which has appeared in the UK over the last 2 years [17]. There is a suggestion that RT 955 is potentially similar to RT027 following detection of a deletion in the toxin regulator and microbiological fingerprinting showing the isolates are very closely related.’

Within paragraph 5 of the introduction:

‘CDI evidently remains an evolving field, and the current concern is that this new circulating ribotype 955 or future strains will lead to increasing cases of severe or fulminant CDI.’.

Within the section Antimicrobial therapy for the first episode of severe or fulminant/life-threatening CDI in the second paragraph under the heading Metronidazole:

‘Clinicians must take note of the C. difficile strain ribotype (955) which has appeared in the UK over the last 2 years.’

