# Supplementary material for: Management of severe and fulminant Clostridioides difficile infection in adults
Source: J Med Microbiol. 2025 Apr 24;74(4):001991. doi: 10.1099/jmm.0.001991 (PMC12022265; doi:10.1099/jmm.0.001991)
Supplement: Uncited Supplementary Material 1. [file jmm-74-01991-s001.pdf]

Management of severe and fulminant *Clostridioides difficile* infection in adults.

Appendices:

Appendix 1:

Unsuccessful therapies

Cadazolid is a quinoxolidinone antibiotic which was developed specifically for treating CDI(1). In 2019 a randomised control trial compared cadazolid to vancomycin with the primary outcome of clinical cure of CDI but did not reach the primary end point of non-inferiority compared with vancomycin(1).

Tolevamer is a polymer that binds to *C. difficile* Toxin A and B and exerts a neutralising effect (2). The aim was to have a non-antibiotic therapeutic agent against *C. difficile* to minimise impact on gut flora(3). Despite promising results in phase II trials(2), phase III trials showed it was not an effective treatment compared with vancomycin and metronidazole and carried an increased risk of hypokalaemia (4).

Appendix 2:

Teicoplanin for severe CDI

Teicoplanin is a Glycopeptide antibiotic effective against Gram positive bacteria (5). NICE guidelines do not recommend Teicoplanin as a treatment option for clostridium difficile reporting that two of the main studies have significant limitations and are at high risk of bias (6). Cost analysis suggests that Teicoplanin offers a cheaper treatment option compared with other antibiotics (6). A prospective observational study reviewed the use of oral teicoplanin compared with oral vancomycin in 287 patients with severe or severe complicated CDI (in cases where ileus was present rectal preparations of both antibiotics were used) (7). The study suggested that patients treated with teicoplanin had a clinical cure rate of 90.7% compared to 79.4% in those treated with vancomycin(7). This study was non-randomised and therefore it may be difficult to extrapolate the results (7). Several studies completed in the 1990s had shown promising results for the treatment of CDI with teicoplanin, however these were before the Ribotype 027 associated outbreaks and therefore less useful in the context of current guidance (8,9). Further randomised control trials are required to review the use of teicoplanin for severe CDI especially in the context of health economics.

Appendix 3:

Cost of CDI management:

The costs associated with the management of CDI are summarised here in table 13.

Treatment	Cost of course of treatment (in GBP)	Reference
Average hospital stay with episode of CDI.	7713.00	Wilcox et al. 2017. (10)
Metronidazole 500mg IV TDS 10 days.	144.30	BNF Metronidazole (11)
Fidaxomicin 200mg oral BD 10 days	1350.00	BNF Fidaxomicin (11)

Teicoplanin 200mg oral BD 10 days	69.00	Drugs and pharmaceutical electronic market information tool (eMIT), 2020 (12)
Vancomycin 125mg oral QDS 10 days	191.83	BNF Vancomycin (11)
Vancomycin 500mg oral QDS 10 days	376.40	BNF Vancomycin (11)
FMT via NGT	740.16	Abdali et al. 2020 (13)
FMT via colonoscopy	3006.17	Abdali et al. 2020 (13)

Table 13 (Appendix 3): Costs associated with management of CDI.

Appendix 4:

Search methods:

Databases searched:

- PubMed (https://pubmed.ncbi.nlm.nih.gov/)
- Cochrane Library (https://www.cochranelibrary.com/)
- Ebsco (https://www.ebsco.com/)

Search terms used:

- Clostridium Difficile
- Clostridium Difficile Infection
- Clostridium difficile AND Severe
- Clostridium difficile infection AND risk factors
- Clostridium difficile AND ribotype
- Clostridium difficile AND outbreak
- Fulminant Clostridium Difficile
- Life threatening Clostridium Difficile
- Antibiotics AND Clostridium Difficile
- Clostridium Difficile AND Scoring AND Severity
- Clostridium Difficile AND ATLAS
- Clostridium Difficile AND surgical management
- Clostridium Difficile AND surgical management AND mortality
- Total Colectomy
- Loop Ileostomy
- Total Colectomy AND Clostridium Difficile
- Loop Ileostomy AND clostridium Difficile
- Surgical candidates
- Surgical Candidates AND Clostridium Difficile.
- Faecal Microbiota Transplant
- Faecal Microbiota Transplant AND Clostridium Difficile.
- Faecal Microbiota Transplant AND Clostridium Difficile AND Severe
- Faecal microbiota transplantation in acute clostridium difficile
- Fidaxomicin

- Fidaxomicin AND Clostridium Difficile
- multidisciplinary team AND clostridium difficile
- Vancomycin
- vancomycin AND clostridium difficile
- vancomycin AND Severe clostridium difficile
- bezlotoxumab
- bezlotoxumab AND clostridium difficile
- intravenous Immunoglobulin clostridium difficile
- Metronidazole AND clostridium difficile
- Metronidazole
- Metronidazole AND severe Clostridium Difficile.
- Tolevamer AND clostridium difficile
- Probiotics AND Clostridium Difficile
- Vendanta
- Teicoplanin
- Cadazolid
- Rifaximin

1. Gerding DN, Cornely OA, Grill S, Kracker H, Marrast AC, Nord CE, et al. Cadazolid for the treatment of Clostridium difficile infection: results of two double-blind, placebo-controlled, non-inferiority, randomised phase 3 trials. Lancet Infect Dis. 2019 Mar;19(3):265–74.
2. Louie TJ, Peppe J, Watt CK, Johnson D, Mohammed R, Dow G, et al. Tolevamer, a novel nonantibiotic polymer, compared with vancomycin in the treatment of mild to moderately severe Clostridium difficile-associated diarrhea. Clin Infect Dis. 2006 Aug 15;43(4):411–20.
3. Musgrave CR, Bookstaver PB, Sutton SS, Miller AD. Use of alternative or adjuvant pharmacologic treatment strategies in the prevention and treatment of Clostridium difficile infection. International Journal of Infectious Diseases. 2011 Jul;15(7):e438–48.
4. Baines SD, Freeman J, Wilcox MH. Tolevamer is not efficacious in the neutralization of cytotoxin in a human gut model of Clostridium difficile infection. Antimicrob Agents Chemother. 2009 May;53(5):2202–4.
5. Ojha SC, Phanchana M, Harnvoravongchai P, Chankhamhaengdecha S, Singhakaew S, Ounjai P, et al. Teicoplanin Suppresses Vegetative Clostridioides difficile and Spore Outgrowth. Antibiotics [Internet]. 2021 Aug 15;10(8):984. Available from: https://www.ncbi.nlm.nih.gov/pmc/articles/PMC8388965/
6. National Institute for Health and Care Excellence (NICE), Public Health England. NICE Guidance. 2021. Clostridioides difficile infection: antimicrobial prescribing. Available from: https://www.nice.org.uk/guidance/ng199
7. Popovic N, Korac M, Nesic Z, Milosevic B, Urosevic A, Jevtovic D, et al. Oral teicoplanin versus oral vancomycin for the treatment of severe Clostridium difficile infection: a prospective observational study. European Journal of Clinical Microbiology & Infectious Diseases [Internet]. 2018 Apr 3;37(4):745–54. Available from: https://link.springer.com/article/10.1007/s10096-017-3169-3

8. Wenisch C, Parschalk B, Hasenhundl M, Hirschl AM, Graninger W. Comparison of Vancomycin, Teicoplanin, Metronidazole, and Fusidic Acid for the Treatment of Clostridium difficile--Associated Diarrhea. Clinical Infectious Diseases. 1996 May 1;22(5):813–8.

9. de Lalla F, Nicolin R, Rinaldi E, Scarpellini P, Rigoli R, Manfrin V, et al. Prospective study of oral teicoplanin versus oral vancomycin for therapy of pseudomembranous colitis and Clostridium difficile-associated diarrhea. Antimicrob Agents Chemother. 1992 Oct;36(10):2192–6.

10. Wilcox MH, Ahir H, Coia JE, Dodgson A, Hopkins S, Llewelyn MJ, et al. Impact of recurrent Clostridium difficile infection: hospitalization and patient quality of life. Journal of Antimicrobial Chemotherapy [Internet]. 2017 Sep 1;72(9):2647–56. Available from: https://academic.oup.com/jac/article/72/9/2647/3867671

11. British Medical Association and Royal Pharmaceutical Society. British National Formulary 87 March - September 2024. 87th ed. London: BMJ Publishing Group Ltd; 2024.

12. Department of Health and Social Care. Gov.UK. 2020. Drugs and pharmaceutical electronic market information tool (eMIT). Available from: https://www.gov.uk/government/publications/drugs-and-pharmaceutical-electronic-market-information-emit

13. Abdali ZI, Roberts TE, Barton P, Hawkey PM. Economic evaluation of Faecal microbiota transplantation compared to antibiotics for the treatment of recurrent Clostridioides difficile infection. EClinicalMedicine [Internet]. 2020 Jul;24:100420. Available from: https://www.ncbi.nlm.nih.gov/pmc/articles/PMC7327885/
